# Artificial intelligence-based pulmonary vessel segmentation: an opportunity for automated three-dimensional planning of lung segmentectomy

**DOI:** 10.1093/icvts/ivaf101

**Published:** 2025-05-19

**Authors:** Quinten J Mank, Abdullah Thabit, Alexander P W M Maat, Sabrina Siregar, Theo van Walsum, Jolanda Kluin, Amir H Sadeghi

**Affiliations:** Department of Cardiothoracic Surgery, Thoraxcenter, Erasmus MC, University Medical Center Rotterdam, Rotterdam, The Netherlands; Biomedical Imaging Group Rotterdam, Department of Radiology & Nuclear Medicine, Erasmus MC, University Medical Center Rotterdam, Rotterdam, The Netherlands; Department of Cardiothoracic Surgery, Thoraxcenter, Erasmus MC, University Medical Center Rotterdam, Rotterdam, The Netherlands; Department of Cardiothoracic Surgery, Thoraxcenter, Erasmus MC, University Medical Center Rotterdam, Rotterdam, The Netherlands; Biomedical Imaging Group Rotterdam, Department of Radiology & Nuclear Medicine, Erasmus MC, University Medical Center Rotterdam, Rotterdam, The Netherlands; Department of Cardiothoracic Surgery, Thoraxcenter, Erasmus MC, University Medical Center Rotterdam, Rotterdam, The Netherlands; Department of Cardiothoracic Surgery, Thoraxcenter, Erasmus MC, University Medical Center Rotterdam, Rotterdam, The Netherlands; Department of Cardiothoracic Surgery, University Medical Center Utrecht, Utrecht, The Netherlands

**Keywords:** cardiothoracic surgery, artificial intelligence (AI), deep learning (DL), pulmonary vessels, lung segmentectomy

## Abstract

**OBJECTIVES:**

This study aimed to develop an automated method for pulmonary artery and vein segmentation in both left and right lungs from computed tomography (CT) images using artificial intelligence (AI). The segmentations were evaluated using PulmoSR software, which provides 3D visualizations of patient-specific anatomy, potentially enhancing a surgeon’s understanding of the lung structure.

**METHODS:**

A dataset of 125 CT scans from lung segmentectomy patients at Erasmus MC was used. Manual annotations for pulmonary arteries and veins were created with 3D Slicer. nnU-Net models were trained for both lungs, assessed using Dice score, sensitivity and specificity. Intraoperative recordings demonstrated clinical applicability. A paired t-test evaluated statistical significance of the differences between automatic and manual segmentations.

**RESULTS:**

The nnU-Net model, trained at full 3D resolution, achieved a mean Dice score between 0.91 and 0.92. The mean sensitivity and specificity were: left artery: 0.86 and 0.99, right artery: 0.84 and 0.99, left vein: 0.85 and 0.99, right vein: 0.85 and 0.99. The automatic method reduced segmentation time from ∼1.5 hours to under 5 minutes. Five cases were evaluated to demonstrate how the segmentations support lung segmentectomy procedures. *P*-values for Dice scores were all below 0.01, indicating statistical significance.

**CONCLUSIONS:**

The nnU-Net models successfully performed automatic segmentation of pulmonary arteries and veins in both lungs. When integrated with visualization tools, these automatic segmentations can enhance preoperative and intraoperative planning by providing detailed 3D views of patients anatomy.

## GRAPHICAL ABSTRACT

**Figure:**
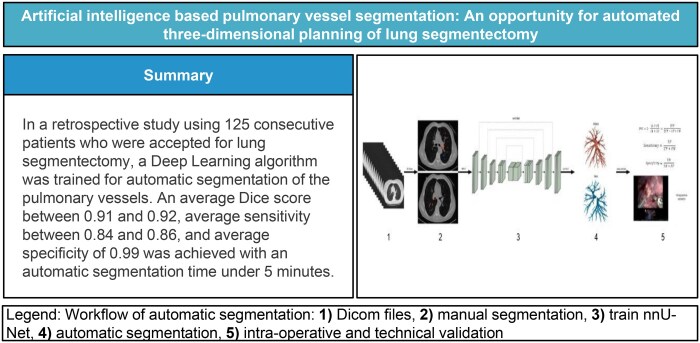

## INTRODUCTION

According to the World Health Organization (WHO), lung cancer is the leading cause of cancer-related death worldwide, with an estimated 2 million deaths in 2023 [1]. Early detection and adequate therapy are essential for patient survival and treatment outcome [2]. Due to an increased application of imaging modalities such as computed tomography (CT), the detection rate of early-stage non-small cell lung cancer (NSCLC) is rapidly increasing [3]. When early-stage NSCLC is diagnosed, it is common practice to perform a lobectomy. However, more recent evidence (e.g. JCOG-0802 trial and CALGB-140503) suggests that for stage 1A1 and stage 1A2 peripherally located lung tumours, a segmentectomy can be a safe alternative approach [4, 5].

While segmentectomy procedures have demonstrated oncological safety, the complexity of performing segmentectomies remains a challenge. Detailed anatomical knowledge of the pulmonary vessels and bronchi is essential in the planning and execution of these resections [6]. Accurate identification of anatomical variations in pulmonary vessels is crucial, particularly to avoid intraoperative bleeding or the inadvertent ligation of vessels during surgery [7]. Currently, conventional two-dimensional (2D) visualization of CT-scans is the golden standard for the planning of anatomical pulmonary resections. However, using CT-slices to detect and classify the relevant anatomy for segmentectomy planning remains challenging, even for experienced surgeons [8]. Using a 2D visualization of the CT scan, the surgeon must mentally create a three-dimensional (3D) representation of the patient’s anatomy and then strive to apply it to the patient’s anatomy during the surgery, adding an extra layer of complexity to the process. Therefore, preoperative 3D-reconstruction of the patient-specific anatomy is valuable and beneficial for segmentectomy planning [7, 9–11], which is also recommended by the European Society of Thoracic Surgeons (ESTS) on technical standards of segmentectomy [12].

The manual reconstruction and segmentation of pulmonary structures can be a solution for achieving 3D visualization of a patient’s anatomy. Various open source or commercially available software, such as 3D Slicer [13] and Materialise Mimics [14], can be leveraged for labelling structures based on pixel intensity. While manual segmentation by a radiologist or an expert reaches high accuracy, it is a time-consuming process, taking up to 1 or several hour(s) [15]. Semi-automatic software tools can reduce processing time but still require interaction by an expert and are often expensive [16].

In recent years, artificial intelligence (AI) has been explored to automate numerous image-processing tasks, including the segmentation of structures of interest [17]. In the literature, numerous publications showcase automated DL-based segmentation methods for pulmonary vessel segmentation. Nonetheless, there is limited literature describing the clinical usability of these models specifically in the context of planning pulmonary segmentectomies. In this study, we utilized and technically evaluated nnU-Net for the automatic segmentation of the pulmonary artery and vein in both the left and right lungs using manually labelled contrast and non-contrast CT scans. Additionally, we have demonstrated the potential clinical usability and feasibility by leveraging a 3D visualization software (PulmoSR, Nieuw Vennep, The Netherlands) in combination with the DL-based segmentations for pulmonary segmentectomy planning.

Our ultimate goal is to provide an automated segmentation tool for the complex task of 3D pulmonary vessels (artery and vein) segmentation which can be used for the preoperative segmentectomy planning process. Such a tool may be used to reduce the preoperative manual workload required for the 3D reconstruction of these structures and provide a better 3D understanding of patient specific anatomy for the surgeon.

## METHODS

### Ethics statement

The study was approved by the Institutional Medical Ethical Committee (MEC-2023–008/MEC-2023–0397). Participants provided written informed consent before participating. CT scans were stored in a pseudonymized way on a secured drive at the Erasmus MC, Rotterdam, The Netherlands.

### Patient population

One hundred twenty-five consecutive patients who were accepted for lung segmentectomy between June 2021 and July 2023 at the Erasmus MC, Rotterdam, The Netherlands, were included in this study after obtaining written informed consent approved. The inclusion criteria were as follows: (i) pulmonary pathology suitable for lung segmentectomy (stage I NSCLC <2 cm, intrapulmonary metastases, and benign lesions limited to lung segments), (ii) age >18 years and (iii) the availability of CT scans with a slice thickness of 1 mm. A total of 120 patient cases were utilized to train and validate the deep learning (DL) models. Additionally, we included five patients who underwent robot-assisted thoracic surgery (RATS) segmentectomy procedures, during which intraoperative recordings were made. These cases were specifically used to demonstrate how the 3D segmentation and visualization provided by our AI models could enhance surgical navigation.

### Segmentation

#### Manual segmentation

The annotation of pulmonary structures was conducted using 3D Slicer (version 4.11), an open-source software designed for medical image visualization and analysis [13]. Each patient’s scan involved annotations of the pulmonary artery and pulmonary vein for both the left and right lung. Despite the presence of pulmonary pathology in one lung, no distinction between the healthy and affected lung was made; both sides were segmented and included in the database for training the DL model. A single operator (QM) performed all segmentations. All segmentations were subsequently verified by a cardiothoracic surgeon (AS) in both 2D (CT and CT-scan overlayed by segmentation) and 3D using a virtual reality-based 3D visualization tool (PulmoVR, Surgical Reality, Nieuw Vennep, The Netherlands) of the CT-scan and segmentation overlay. The manual segmentation process remained uniform across all patients.

#### Automatic segmentation

**Figure 1:**
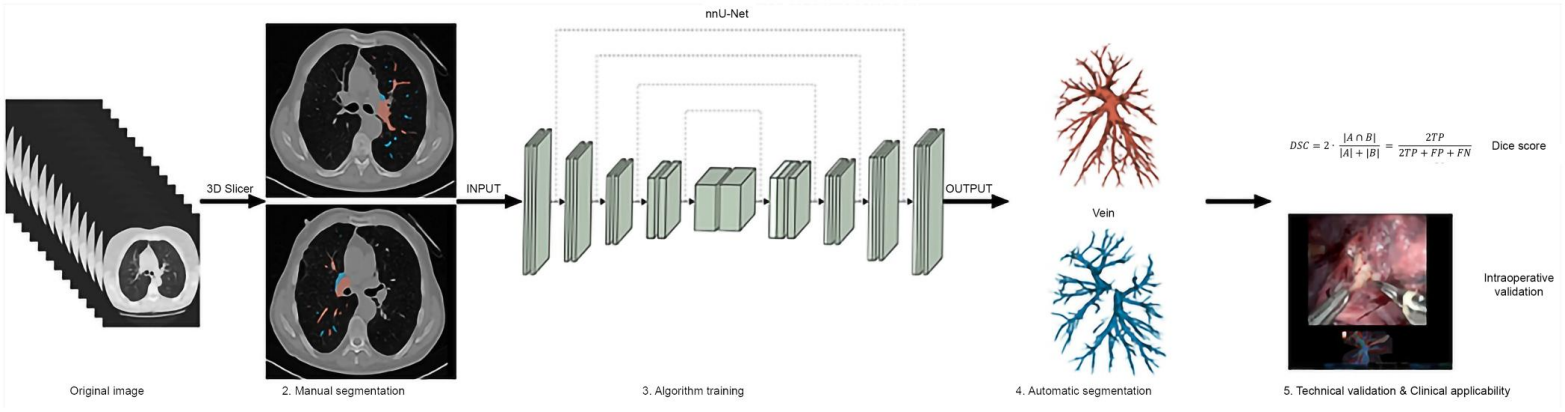
Workflow diagram of the DL-based pulmonary vessel segmentation

nnU-Net was used for the automatic segmentation [18]. The nnU-Net automatically configures itself and executes all the steps in the segmentation pipeline. The architecture of the nnU-Net (Fig. 1) is based on the U-Net, which is a convolutional neural network (CNN) architecture that was proposed for biomedical image segmentation. A dataset of 100 manually segmented arteries and veins of both right and left lung was used as input for training the nnU-Net. Four different models were trained for all structures (left pulmonary artery, left pulmonary vein, right pulmonary artery, right pulmonary vein). After training the nnU-Net, technical evaluation was performed.

For training and validation, a distribution of 80% (80 CT scans) and 20% (20 CT scans) of the dataset was used. An additional 25 CT-scans were used as a separate test set to evaluate the technical (20 scans) and clinical (5 scans and surgical videos) usability of the models.

### Technical validation

To assess the disparity between automatic and manual segmentation techniques, the differences were visualized utilizing 3D Slicer software. Furthermore, the mean segmentation time of the automatic approach was determined. To quantitatively assess the performance of the DL segmentation algorithm, the Dice score (DSC) was used. The DSC measures the overlap between the predicted segmentation and the ground truth manual segmentation. It quantifies how much the predicted region matches the true region in terms of spatial location and size. The DSC is defined as the ratio between true positive (TP), false positive (FP) and false negative (FN) (Equation 1). The DSC ranges from 0 (indicating no overlap between the two segmentation maps) to 1 (reflecting complete overlap between the two segmentation maps).


(1) $$\mathrm{DSC}=2\cdot \frac{\left|A\cap B\right|}{\left|A|+|B\right|}=\frac{2TP}{2TP+FP+FN}$$

where A ∩ B represents the number of pixels that are correctly classified as positive (foreground) in both the predicted mask (A) and the ground truth mask (B). Essentially, it denotes the number of pixels of the overlap between the two masks.

In addition to the DSC, sensitivity and specificity were calculated to provide a more comprehensive evaluation of the model’s performance (Equations 2 and 3). Sensitivity measures the proportion of actual positive pixels (pulmonary vessels) that are correctly identified by the model, while specificity measures the proportion of actual negative pixels (non-vessel regions) that are correctly classified.


(2) $$\mathrm{Sensitivity}=\frac{TP}{TP+FN}$$

(3) $$\mathrm{Specificity}=\frac{TN}{TN+FP}$$

These metrics offer insight into the model’s ability to correctly identify both the vessel and non-vessel regions, which is crucial for assessing both the accuracy and reliability of the segmentation approach.

### Statistical testing

A paired t-test was performed to assess the statistical significance of the differences between the DSCs of the DL segmentation algorithm and the manual segmentation. The significance level (alpha) was set to 0.01. Using the TTestPower function from the statsmodels library in Python, ensuring that the study was designed to achieve a statistical power of 0.9. This power level corresponds to a 90% probability of detecting a true effect if it exists, minimizing the risk of Type II errors and ensuring the robustness of the test.

The reference DSC of 0.869 was derived from relevant literature on pulmonary vessel segmentation performance in similar contexts [19–21]. This value served as a baseline to assess the efficacy of the segmentation model against established standards.

In addition to hypothesis testing, the 95% confidence interval (CI) for the average DSCs in the test set was calculated. The CI provides a range within which the true mean DSC is likely to fall, offering a measure of the precision and reliability of our segmentation results. This CI was also used to compare our results with the reference DSC, further validating the performance of our model.

### Clinical applicability

To demonstrate feasibility, we have retrospectively analysed the model’s performance in five patients who underwent robot-assisted pulmonary segmentectomy. To describe the clinical usability of the 3D models, an additional application of the use of 3D segmentations was illustrated. By loading the automatic segmentations in PulmoSR software (Surgical Reality, Nieuw Vennep, The Netherlands), a deformable 3D model can be generated. Manual deformation of the 3D model enables simulation (e.g. deformation of lung lobes, such as posterior retraction or opening the fissure) of realistic intraoperative scenarios [22]. Utilizing intraoperative recordings, anatomical verification can be performed by comparing the deformable 3D model, derived from automatic segmentation, with intraoperative situations where the lung anatomy (artery, vein and bronchus) was observable.

## RESULTS

### Comparison between manual and automatic segmentation

**Figure 2:**
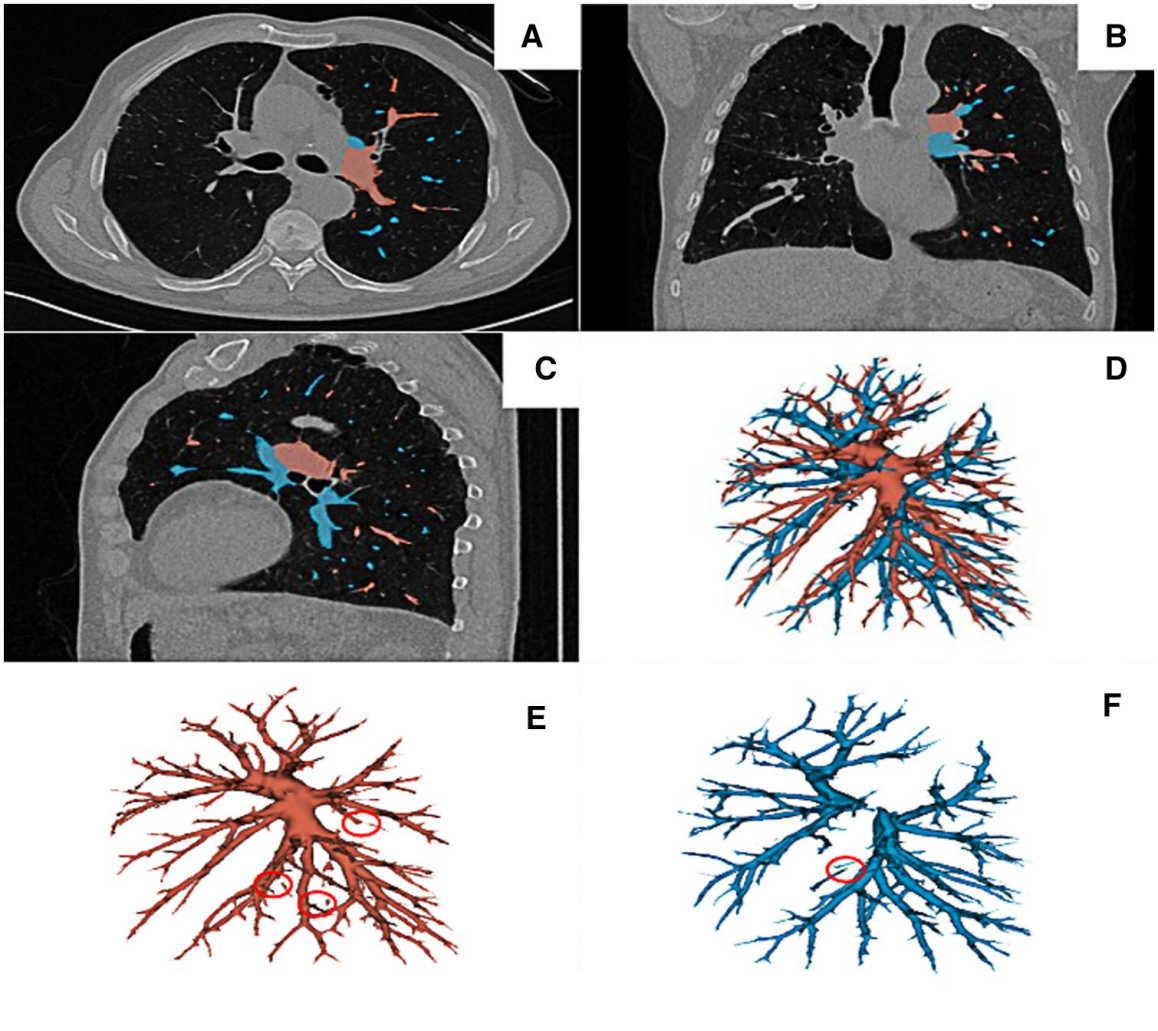
Overlap of automated segmentation of the left pulmonary artery (red) and the left pulmonary vein (blue) in axial view (**A**), coronal view (**B**) and sagittal view (**C**) on top of the CT scan. Full 3D visualization of the segmentation (**D**). Missing parts in the automatic segmentation for the left pulmonary artery (**E**) and left pulmonary vein (**F**) are highlighted with the red circles

A 3D visualization of the left pulmonary vessels was generated together with 2D CT scans with a coloured segmentation in Fig. 2. Supplementary Figure S1 provides a 3D visualization of the right pulmonary vessels.

**Figure 3:**
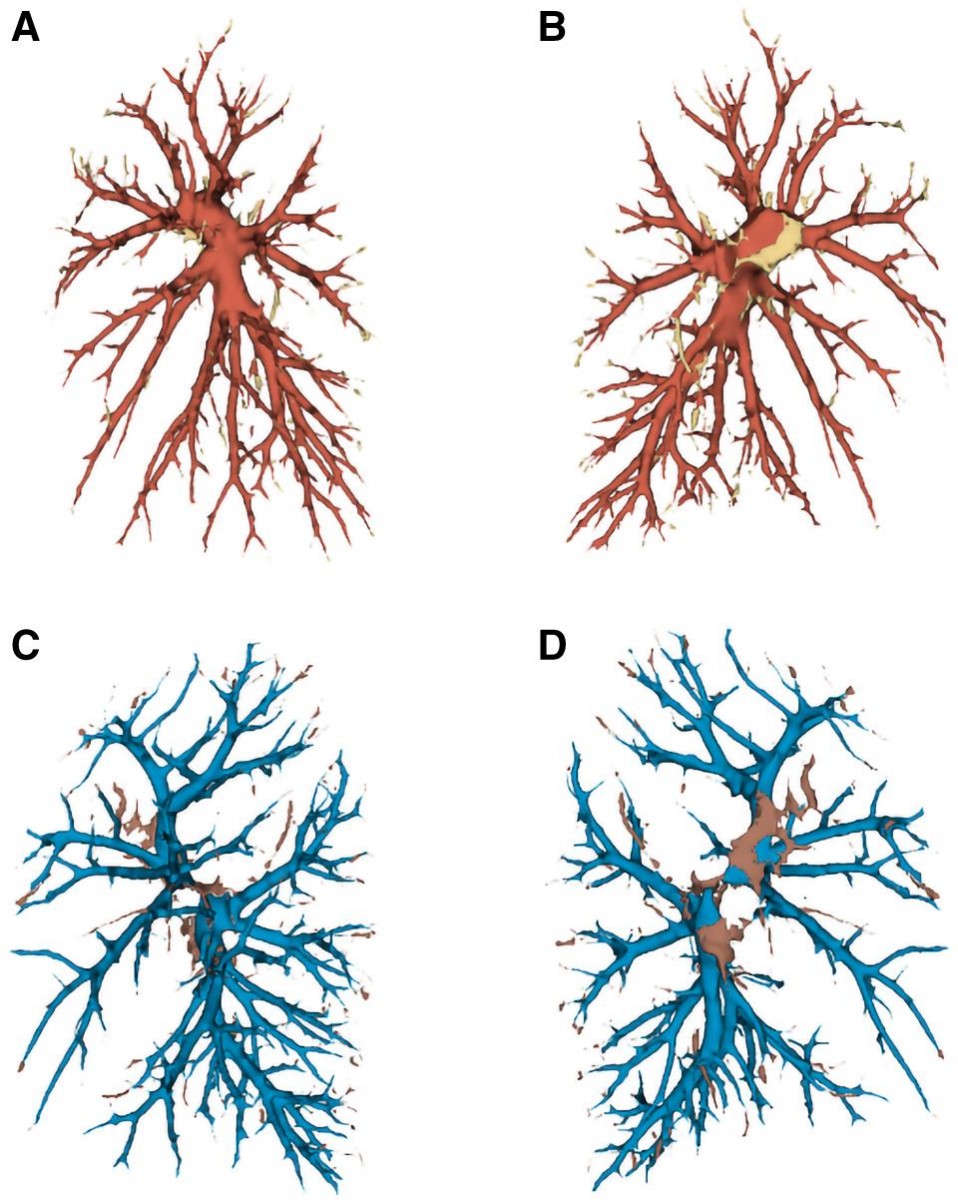
Automatic segmentation of the left pulmonary artery (red, **A** and **B**) and the left pulmonary vein (blue, **C** and **D**). The difference with the manual segmentation is coloured in yellow (pulmonary artery, **A** and **B**), and brown (pulmonary vein, **C** and **D**)

Figure 3 shows that the difference between the left manual and automatic segmentation is most prominent at the hilum of the heart and the most peripheral branches of the vessels. Supplementary Figure S2 provides the difference between the right manual and automatic segmentation.

The mean segmentation time for all structures (right artery, right vein, left artery and left vein) was as follows: 147 seconds (left artery), 137 seconds (right artery), 142 seconds (left vein) and 139 seconds (right vein).

### Technical validation

**Figure 4:**
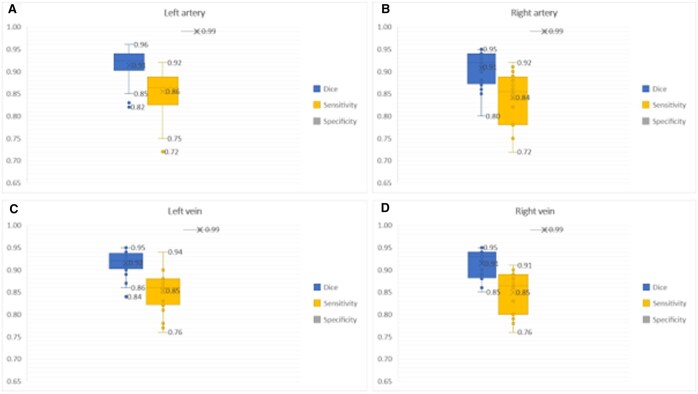
Technical validation on the evaluation set of 20 CT scans using Dice score, sensitivity and specificity. The maximum score, minimum score, mean score (=X) and outliers are presented using a box and whisker plot for left artery (**A**), right artery (**B**), left vein (**C**) and right vein (**D**)

The performance scores for the evaluation metrics are presented in Fig. 4.

A mean DSC of 0.91 is observed for the left artery (95% CI: 0.90–0.93), right artery (95% CI: 0.89–0.93) and right vein (95% CI: 0.90–0.93), while the left vein showed an mean DSC of 0.92 (95% CI: 0.90–0.93). The mean sensitivity and specificity are respectively: left artery: 0.86 and 0.99, right artery: 0.84 and 0.99, left vein: 0.85 and 0.99, right vein: 0.85 and 0.99.

### Statistical testing

The results of the significance level (alpha) calculation for the DSCs demonstrated statistically significant differences for all vessel categories, with alpha values below the threshold of 0.01. The calculated *P*-values for the DSCs were as follows: artery left: 0.0014, artery right: 0.0096, vein left: 0.00048 and vein right: 0.000105.

### Clinical applicability

**Figure 5:**
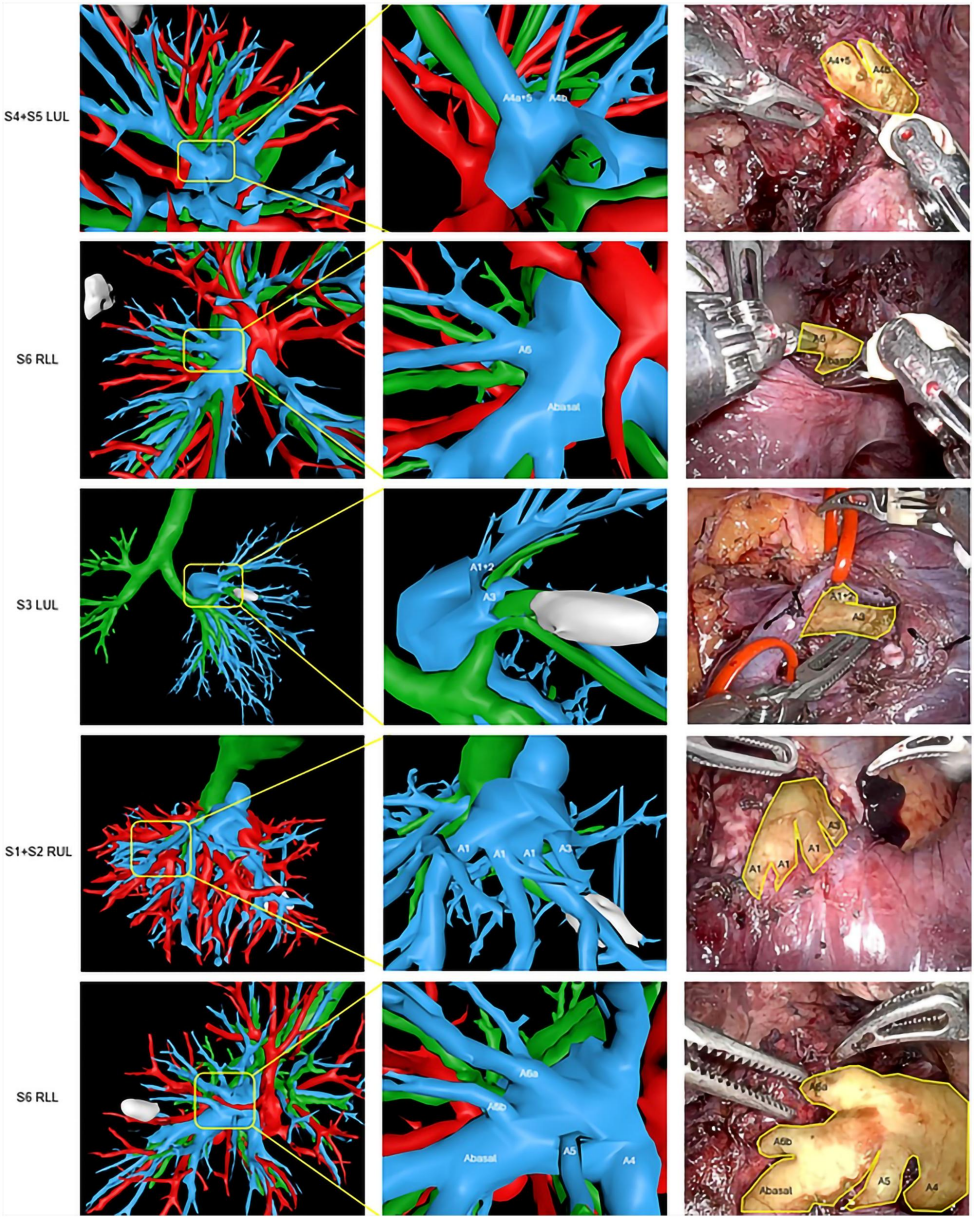
Clinical applicability of the pulmonary artery using five RATS segmentectomy procedures (first column indicates the resected segment) with an overview of the automatic segmentation (artery = blue, vein = red, airways = green) visualized as a 3D model utilizing PulmoSR software (second column), zoom in (third column) and intraoperative situation (fourth column). LUL: left upper lobe; RLL: right lower lobe; RUL: right upper lobe

As illustrated in Figs 5 (pulmonary artery) and 6 (pulmonary vein), the intraoperative vasculature of five RATS segmentectomy patients was visualized using the automatic segmentations. By using intraoperative recordings in combination with the dynamic 3D model, realistic situations of the procedure can be simulated after manually deforming the 3D model by a surgeon to match the corresponding anatomical orientation in the patient.

**Figure 6:**
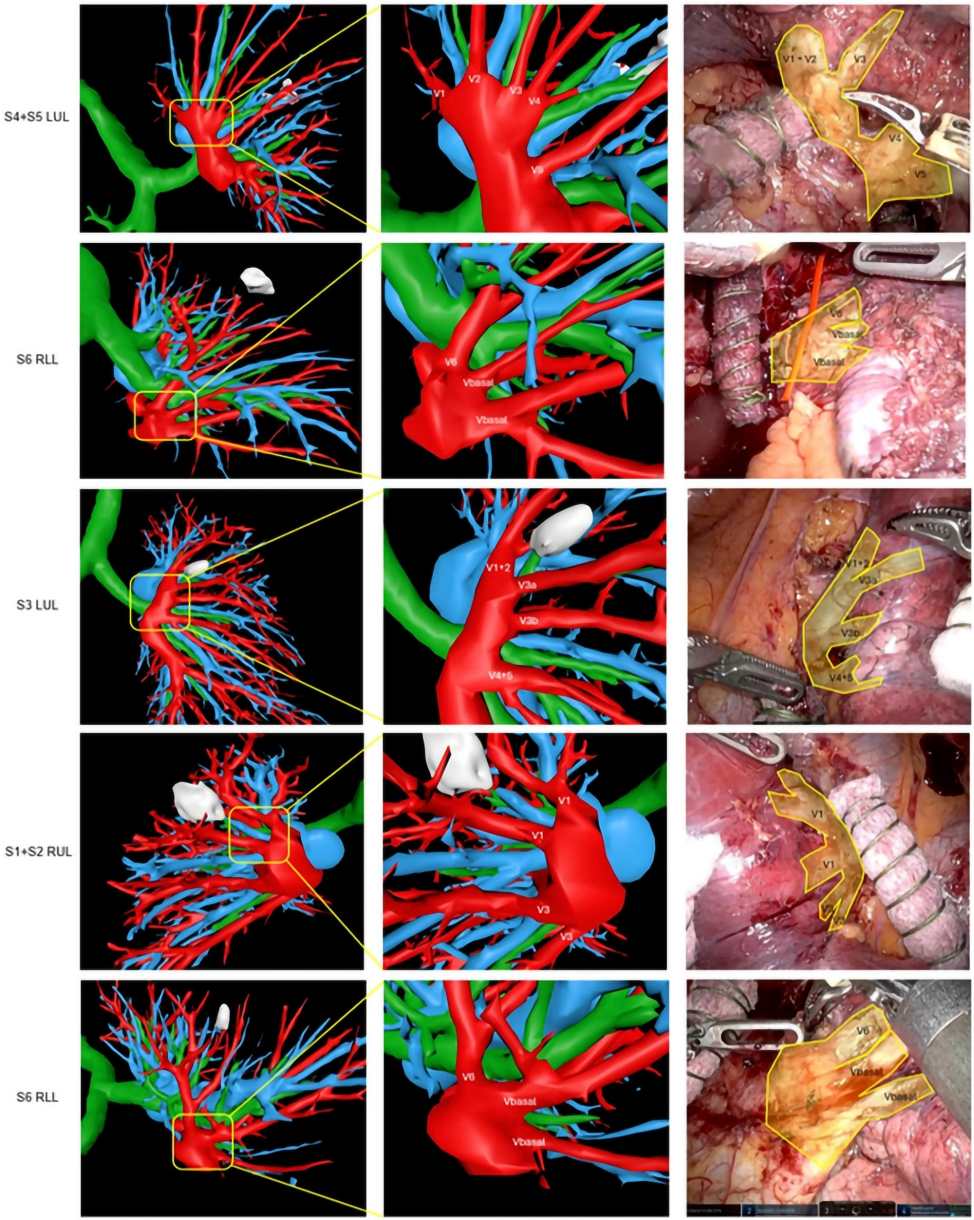
Clinical applicability of the pulmonary vein using five RATS segmentectomy procedures (first column indicates the resected segment) with an overview of the automatic segmentation (artery = blue, vein = red, airways = green) visualized as a 3D model utilizing PulmoSR software (second column), zoom in (third column) and intraoperative situation (fourth column). LUL: left upper lobe; RLL: right lower lobe; RUL: right upper lobe

## DISCUSSION

In this study, we developed and evaluated four nnU-Net models for fast and accurate 3D segmentation of pulmonary arteries and veins using a dataset of 100 manually annotated CT images. The nnU-Net models achieved fully automatic pulmonary vessel segmentation within 5 minutes, offering a significant improvement in efficiency compared to traditional semi-automatic methods, which are time-consuming and labor-intensive.

Various studies have demonstrated that the segmentation of the pulmonary artery, pulmonary vein and bronchi improves preoperative planning through 3D visualization [23]. Previously, our group has demonstrated the added value of (virtual reality-based) 3D visualization in the planning of segmentectomy procedures [7, 11]. In these studies, a significant ±50% change of original surgical plan (which was based on 2D-CT) was observed when 3D-based planning was additionally applied. Even though in these studies the intersegmental borders were automatically segmented using Thirona’s (Nijmegen, The Netherlands) LungQ AI-based software [24], the visualization of the pulmonary vessels and bronchi was created through semi-automatic (partly manual) segmentation. In order to decrease the segmentation workload and increase the accuracy of 3D planning, we have successfully trained nnU-Net models for automatic segmentation of the pulmonary vessels.

The performance of a DL algorithm can be affected by the quality and quantity of the data. Therefore, for further research, more CT scans from different institutes must be included to obtain a generalizable and robust DL model [25]. Cui *et al.* achieved a DSC of 0.93 for pulmonary vessel segmentation using a dataset of 300 CT scans [26]. Not only is the size of size of the dataset important to train a DL-model, but the variety of the data also influences the DL model performance. AI models need to be robust and perform well across a variety of data [27]. This study only includes data from our institution, so the performance of the model on external datasets has not been evaluated [27].

The low *P*-values indicate that the differences in DSCs between the predicted and manual segmentations are not due to chance, confirming the robustness of the segmentation model in all vessel categories. These findings underscore the reliability and statistical significance of the DL model in accurately segmenting the pulmonary arteries and veins, which is crucial for applications such as surgical planning and clinical decision-making. Despite these challenges, the statistical significance of the results suggests that our DL-based segmentation model is robust and capable of achieving reliable performance in the automatic segmentation of pulmonary vessels, irrespective of these anatomical and imaging complexities. Future work could focus on further improving the model’s handling of these variabilities, especially in more difficult cases involving non-contrast CT scans or patients with anatomical anomalies.

Some variability was observed, with outliers in the left artery (AL) and left vein (VL) DSCs and sensitivity, due to suboptimal CT images, particularly non-contrast scans. These scans posed challenges for the model due to reduced contrast between vessels and tissues. Additionally, heart motion could have affected thoracic region stability, contributing to segmentation discrepancies. These limitations underscore the importance of high-quality imaging for optimal results. The increased variability observed in the right artery (AR) model, evidenced by a lower minimum DSC, can be attributed to anatomical variations in the right pulmonary artery. The branching patterns of the right pulmonary artery, along with variations in where the truncus anterior branches off from the main pulmonary artery, introduced additional complexity to the segmentation task. In manual segmentation, it was often necessary to include a larger portion of the hilum to ensure complete connectivity, which could influence the model's performance. In particular, non-contrast CT scans exacerbated the problem by making it more difficult to distinguish between the pulmonary artery and surrounding tissues. Consequently, the right artery and vein segmentations exhibited increased variability compared to the left side, further highlighting the challenges presented by anatomical complexity and imaging limitations.

Commonly, DL-based algorithms are evaluated using various technical performance metrics (such as DSC). However, technical evaluations do not necessarily reflect the suitability for clinical use. Each medical application of DL has different accuracy requirements which should be based on the medical tasks that it needs to perform [28]. For lung segmentectomy planning, specific (e.g. hilar and arterial branches) structure detection, especially at the segmental and lobar levels are of higher importance than other peripheral branches (for instance the second or third degree arterial or venous subsegmental branches). Therefore, if these critical structures are segmented correctly, the details in the segmented sub branches are not of utmost importance for performing the resection, even though they do play an important role in defining intersegmental planes.

This study conducted a preliminary clinical usability description utilizing five intraoperative recordings of segmentectomy procedures. However, the scope of this clinical usability description is limited to the visual structures observed during the specific procedures. Segmentation errors may occur in segments of the complete vessel segmentation that are not intraoperatively visualized. We are currently designing a multicentre clinical validation trial to investigate the clinical usability and validity of the algorithm.

Vessel segmentation poses challenges due to similar voxel intensities and cross sections between arteries and veins, making model training and prediction difficult. In our segmentation task, in some cases unconnected and missing elements in the segmentation output were visible (Figs 2 and 3, Supplementary Figs S1 and S2). Various techniques have been proposed to address this issue by reconnecting these parts and filling the missing links. Guo *et al.* have detailed a method for hepatic vascular segmentation and the linkage of fractured portions in the segmentation [29]. Adding this postprocessing step may result in the segmentation of a fully connected vessel tree.

## CONCLUSION

In this study, we successfully trained and validated a nnU-Net-based framework for the automatic segmentation of pulmonary arteries and veins in both left and right lungs using manually annotated CT scans. By integrating the automatic segmentation with 3D visualization software, we demonstrated the potential clinical utility of 3D models for planning and intraoperative guidance in five segmentectomy cases.

In conclusion, our method achieves fully automatic pulmonary artery and vein segmentation within 5 minutes, offering a significant improvement in efficiency that could assist surgeons in the pre- and intraoperative guidance of complex lung segmentectomies. While the current results demonstrate the potential for clinical application in segmentectomy planning, further validation on larger datasets and in diverse clinical settings is needed to confirm the robustness and generalizability of the method. Future work will also focus on improving the model's performance in challenging cases, such as non-contrast CT scans or patients with anatomical anomalies, to further enhance the clinical applicability.

## Supplementary Material

ivaf101_Supplementary_Data

## Data Availability

The data underlying this article will be shared on reasonable request to the corresponding author.

## ABBREVIATIONS

2D: Two-dimensional
3D: Three-dimensional
AI: Artificial intelligence
CI: Confidence interval
CNN: Convolutional neural network
CT: Computed tomography
DSC: Dice score
ESTS: European Society of Thoracic Surgery
FN: False negative
FP: False positive
NSCLC: Non-small cell lung cancer
RATS: Robot-assisted thoracic surgery
TN: True negative
TP: True positive
WHO: World Health Organization
