# Supplementary figures and images for: Artificial intelligence-based pulmonary vessel segmentation: an opportunity for automated three-dimensional planning of lung segmentectomy
Source: Interdiscip Cardiovasc Thorac Surg. 2025 May 19;40(5):ivaf101. doi: 10.1093/icvts/ivaf101 (PMC12103915; doi:10.1093/icvts/ivaf101)

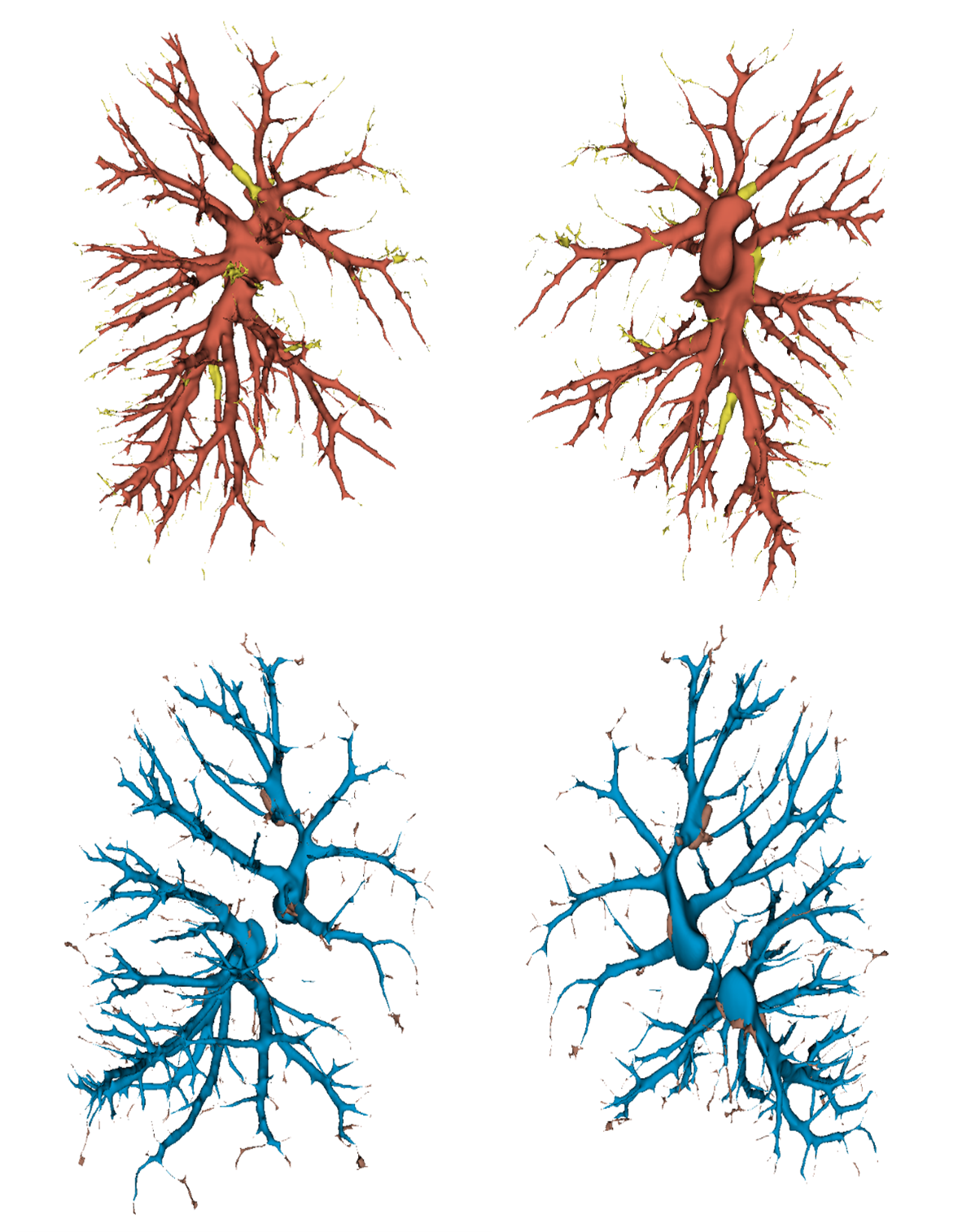

Supplement: ivaf101_Supplementary_Data [file ivaf101_supplementary_data.zip › Supplementary Fig S2.tif]

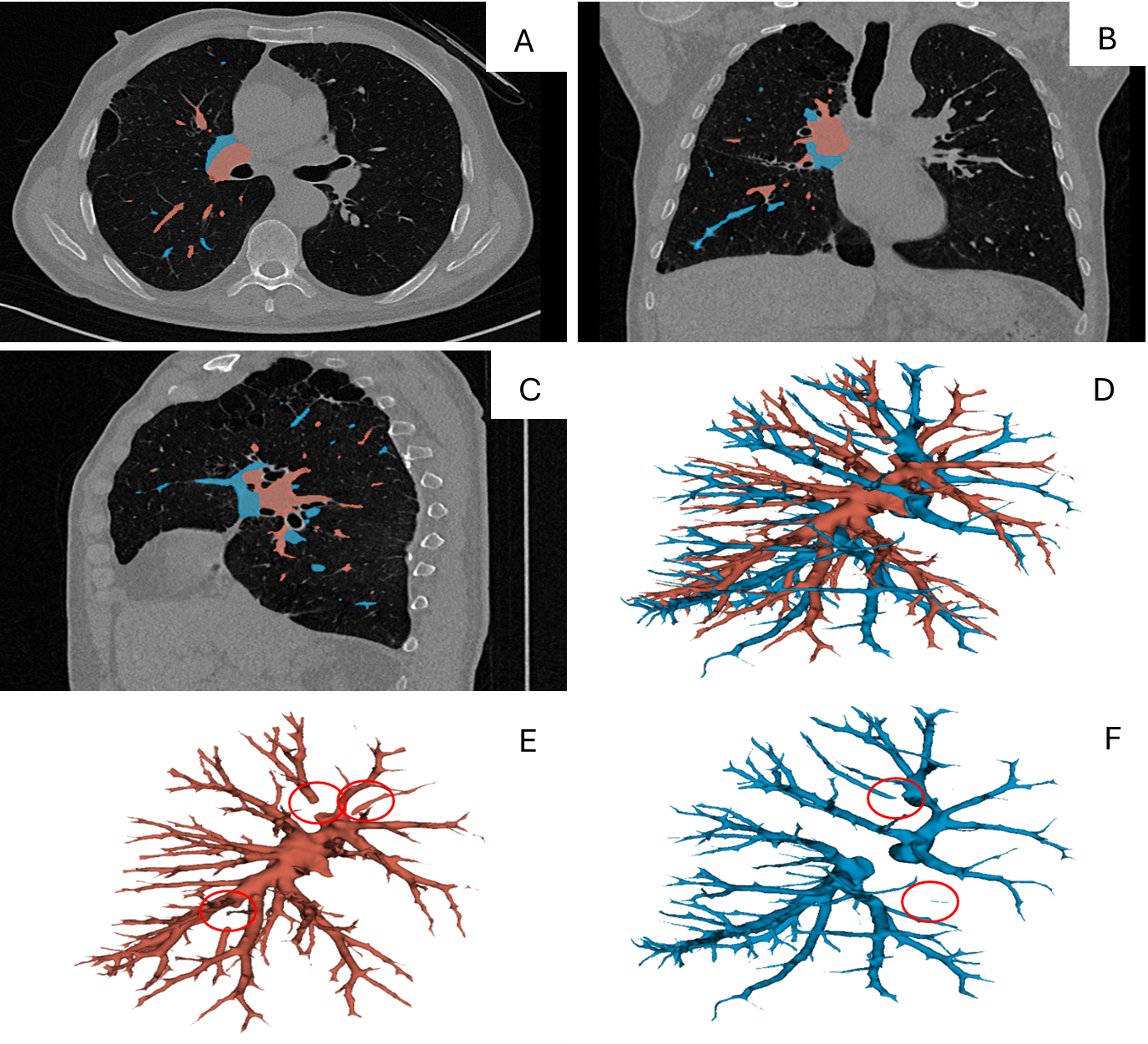

Supplement: ivaf101_Supplementary_Data [file ivaf101_supplementary_data.zip › Supplementary Fig S1.tif]
